# Comparative and genetic analysis of the four sequenced *Paenibacillus polymyxa* genomes reveals a diverse metabolism and conservation of genes relevant to plant-growth promotion and competitiveness

**DOI:** 10.1186/1471-2164-15-851

**Published:** 2014-10-03

**Authors:** Alexander W Eastman, David E Heinrichs, Ze-Chun Yuan

**Affiliations:** Southern Crop Protection & Food Research Centre, Agriculture & Agri-Food Canada, 1391 Sandford Street, London, Ontario N5V 4 T3 Canada; Department of Microbiology & Immunology, The University of Western Ontario, 1151 Richmond St, London, Ontario N6A 5C1 Canada

## Abstract

**Background:**

Members of the genus *Paenibacillus* are important plant growth-promoting rhizobacteria that can serve as bio-reactors. *Paenibacillus polymyxa* promotes the growth of a variety of economically important crops. Our lab recently completed the genome sequence of *Paenibacillus polymyxa* CR1. As of January 2014, four *P. polymyxa* genomes have been completely sequenced but no comparative genomic analyses have been reported.

**Results:**

Here we report the comparative and genetic analyses of four sequenced *P. polymyxa* genomes, which revealed a significantly conserved core genome. Complex metabolic pathways and regulatory networks were highly conserved and allow *P. polymyxa* to rapidly respond to dynamic environmental cues. Genes responsible for phytohormone synthesis, phosphate solubilization, iron acquisition, transcriptional regulation, σ-factors, stress responses, transporters and biomass degradation were well conserved, indicating an intimate association with plant hosts and the rhizosphere niche. In addition, genes responsible for antimicrobial resistance and non-ribosomal peptide/polyketide synthesis are present in both the core and accessory genome of each strain. Comparative analyses also reveal variations in the accessory genome, including large plasmids present in strains M1 and SC2. Furthermore, a considerable number of strain-specific genes and genomic islands are irregularly distributed throughout each genome. Although a variety of plant-growth promoting traits are encoded by all strains, only *P. polymyxa* CR1 encodes the unique nitrogen fixation cluster found in other *Paenibacillus* sp.

**Conclusions:**

Our study revealed that genomic loci relevant to host interaction and ecological fitness are highly conserved within the *P. polymyxa* genomes analysed, despite variations in the accessory genome. This work suggets that plant-growth promotion by *P. polymyxa* is mediated largely through phytohormone production, increased nutrient availability and bio-control mechanisms. This study provides an in-depth understanding of the genome architecture of this species, thus facilitating future genetic engineering and applications in agriculture, industry and medicine. Furthermore, this study highlights the current gap in our understanding of complex plant biomass metabolism in Gram-positive bacteria.

**Electronic supplementary material:**

The online version of this article (doi:10.1186/1471-2164-15-851) contains supplementary material, which is available to authorized users.

## Background

Plant-growth promoting rhizobacteria (PGPR) increase the fitness and growth of host plants through various mechanisms, including biological nitrogen fixation, synthesis of plant hormones, solubilization of inorganic mineral phosphates, and siderophore production [1–5]. Many PGPR prevent colonization of the rhizosphere by pathogenic or parasitic organisms through both direct and indirect mechanisms. Direct inhibition of pathogens typically involves secretion of antagonistic compounds and/or direct lysis mechanisms [6–8], while indirect mechanisms may include induction of plant defenses [9] and/or competition for nutrients, such as iron, that are limited in the soil environment [1, 10]. The Gram-positive, sporulating, facultative anaerobic *Paenibacillus* genus is comprised of over 150 species of which many act as important PGPRs in agriculture and horticulture [11]. *Paenibacillus polymyxa* is a well-studied PGPR and strains have been isolated from diverse geographic regions and ecological niches [12–18]. *P. polymyxa* was originally identified and characterized as *Bacillus polymyxa* as a result of its similarity to *Bacillus* sp. [19]. *P. polymyxa* strains are best known for their production of various potent anti-microbial and volatile compounds that improve plant fitness through nutrient cycling, pathogen antagonism and induction of plant defenses [9–11, 20–25]. Recent interest in the species has been driven by agribusiness and government initiatives for applications in bio-control, bio-fertilization and bio-fuel production [26].

Recently, our lab isolated *P. polymyxa* CR1 (hence forth CR1) from the rhizosphere of degrading corn stalks in southern Ontario, Canada. CR1 produces large amounts of indole-3-acetic acid (IAA) and excretes antagonistic compounds active against several plant pathogenic bacteria and fungi (unpublished data). Additionally*,* CR1 was able to fix-nitrogen and promote the growth of a variety of crops. Interestingly, CR1 metabolized lignin, cellulose and hemi-cellulose as a sole carbon source, and fermented these plant-derived compounds directly into alcoholic compounds (Weselowski *et al.*, manuscript in preparation). To better understand the relevant *P. polymyxa* CR1 metabolic pathways and regulatory mechanisms, we sequenced, assembled and annotated the CR1 genome [27]. As of January 2014, four *P. polymyxa* genomes have been completely sequenced and annotated [27–30]: *P. polymyxa* E681, *P. polymyxa* M1, *P. polymyxa SC2,* and *P. polymyxa* CR1 (hence forth E681, M1, SC2, and CR1, respectively).

E681, isolated in Korea from the rhizosphere of barley, was the first *P. polymyxa* strain to be completely sequenced. It promotes plant growth through production of indole-3-acetic acid (IAA); and produces volatile compounds that elicit induced-systemic resistance in plants, priming the plant host defenses for a rapid response against pathogenic bacteria [22, 31]. E681 has been investigated for various agricultural applications including microbial fertilization and bio-control activities against economically significant plant pathogens [20, 32]. Both M1 and SC2 were isolated in China from wheat roots and pepper rhizosphere respectively. All studied *P. polymyxa* strains possess various non-ribosomal peptide synthetase gene clusters encoding polymyxins, fusaricidins, paenicidins, gramicidins, bacitracins, polyketides, a bacilorin and a lantibiotic [6, 10, 29]. All strains of *P. polymyxa* are capable of strong growth antagonism of various bacterial and fungal plant pathogens and parasitic nematodes [9, 10, 14, 20, 21, 23–25, 32]. Furthermore, siderophores encoded by *P. polymyxa* increase plant-growth in iron-limited conditions by increasing iron availability for the associated plant host and decreasing iron availability for pathogenic organisms [33].

As a major feature of the post-genomic era in biology, comparative genomics is recognized as an important tool for identifying and dissecting key metabolic pathways and regulatory networks amongst related organisms [34–37]. These analyses provide insights into genetic features that have been acquired, modified, or lost during adaptation to specific environmental niches [38–40]. In particular, *in silico* comparisons and analyses of taxonomically related bacterial species allows for rapid identification of functional similarities and unique genetic elements, uncovering complicated metabolic pathways and regulatory networks in the process [38, 40–42]. Despite its potential importance in agriculture and industry, no comparative genomic studies have been completed for *P. polymyxa*. Limited information is available regarding the specific adaptations and mechanisms that allow for their survival and growth in the soil environment.

Here we perform detailed comparative genome analyses of the four sequenced *P. polymyxa* strains. The genomes of each strain were screened for the presence of loci associated with plant growth promotion and disease control. We also compared genomic regions implicated in metabolic versatility and association with plant hosts. These comparative genomic analyses have expanded our understanding of *P. polymyxa* biology and highlighted the gap in our current understanding of biomass metabolism pathways in Gram-positive bacteria. This work will provide a foundation for follow-up studies of target genes and functions and will facilitate genetic and metabolic engineering of *P. polymyxa* to improve agricultural and industrial applications.

## Results and Discussion

### Phylogenetic analyses

**Figure 1:**
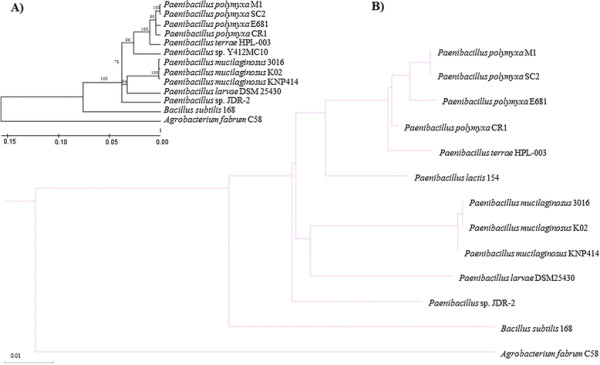
**16S rRNA maximum-likelihood phylogenetic tree of completed sequenced** ***Paenibacillus*** **sp** ***.*** **A)** Sequences of complete genomes were obtained from NCBI Genebank. The phylogenetic tree was generated in MEGA6 using the maximum-likelihood method with 1000 bootstrap replications. Numbers at each branch point correspond to the proportion of positive results from bootstrapping. **B)** Neighbour joining whole-genome phylogeny generated using the dnadist and neighbour packages in PHYLIP and visualized using phyloXML. Branch lengths are representative of the number of nucleotide substitutions per site. *Agrobacterium fabrum* C58 was used as an out-group, while *Bacillus subtilis* 168 was included to corroborate the previously reported close relationship to *Paenibacillus* sp*.*

A phylogenetic tree was generated based on the 16S rRNA of completely sequenced *Paenibacillus* sp. using the Maximum-likelihood method [43] in MEGA6 [44] (Figure 1, panel A). To support the obtained 16S rRNA phylogeny, a whole-genome neighbour-joining phylogenetic tree was generated using the dnadist and neighbour packages in Phylip and visualized using PhyloXML (Figure 1, panel B). On the basis of a 16S rDNA phylogeny, CR1 and E681 form their own clade while M1 and SC2 form a separate clade. However, when the whole genome is used to compute the phylogeny, CR1 and E681 are no longer in the same clade. Our results suggest *P. polymyxa* is most distantly related to *Paenibacillus mucilaginosus*, which is currently employed in microbial fertilizers [45], while being most closely related to *Paenibacillus terrae*, a diazotrophic, free-living soil bacterium.

### General genomic features

General features of the four completely sequenced *Paenibacillus polymyxa* genomes are presented in Table 1. Immediately evident is the variation in genome size and differences in plasmid content amongst the strains, with no plasmids present in CR1 or E681. Genome size varies between 5.3 Mb in E681 to 6.02 Mb in CR1 excluding plasmids, with chromosome coding DNA sequences (CDS) varying between 4805 and 5406, respectively. The mean G + C mol% of the species is 45.4% and no strain deviates from the mean by >0.6%. The plasmids of SC2 and M1 have a lower G + C mol% in accordance with previously reported research [46]. With the inclusion of plasmids in the calculation, SC2 has the largest genome and the most CDS (6.24 Mb, 6020 CDS) with M1 in a close second in terms of size, albeit with significantly fewer CDS (6.23 Mb, 5342 CDS). This difference of 678 CDS between M1 and SC2 is remarkable considering the genome size only varies by approximately 10 kb between the two strains (Tables 1 and 2). The differences in CDS between M1 and SC2 is likely the result of different annotation methods employed, which can result in large discrepancies in the total number of genes identified in a genome [47]. Despite the absence of a plasmid, CR1 has a large genome size (6024 kb), likely resulting from the large number of horizontally acquired genes presented later.

**Table 1:** **General genome features of completely sequenced** ***Paenibacillus polymyxa*** **strains**

	CR1	E681	SC2	M1
Accession numbers	NC_023037	NC_014483	NC_014622	NC_017542
Location of isolation (Country)	Corn Rhizosphere (Canada)	Barley Rhizosphere (Korea)	Pepper Rhizosphere (China)	Wheat Roots (China)
Genome size (base pairs)	6 024 666	5 394 884	5 731 816	5 864 546
GC content (%)	45.6	45.8	45.2	44.8
Protein-coding genes	5 283	4 805	5 406	5 069
Plasmid size (base pairs)	NA	NA	510 115	366 576
Pseudogenes	103	1	52	
rRNA genes	36	36	42	42
tRNA genes	87	91	110	110
Other RNA genes	1			
Conserved CDS	3463	3457	3505	3338
Strain Specific CDS	955	443	11	121

NA, not applicable, refers to strains in which no plasmids are naturally present. Accession numbers refer to the genome sequence entry in NCBI Nucleotide database. Coding sequences, pseudogenes, and RNA genes were identified from available annotations in the NCBI Genebank database. tRNA and rRNA genes were re-identified using tRNAscan and RNAmmer respectively. Conserved and strain-specific sequences were determined using mGenomeSubtractor, with an H-value cut-off 0.81 and 0.41 respectively.

**Table 2:** **Plasmid features of** ***P. polymyxa strains*** **SC2 and M1**

	SC2	M1
Accession Number	NC_014628	NC_017543
Plasmid Size (base pairs (GC%))	510 115 (37.6)	366 576 (38.4)
Plasmid Genes (Protein, tRNA)	676 (626, 44)	295 (295, 0)
Strain Specific CDS	614	273

Accession numbers to the plasmid entry in NCBI Nucleotide database. Coding Sequences were identified from available annotations in the NCBI Genebank database. Conserved and strain-specific plasmid encoded sequences were determined using mGenomeSubtractor, with an H-value cut-off of 0.81 and 0.41 respectively.

### Conservation of genome architecture

**Figure 2:**
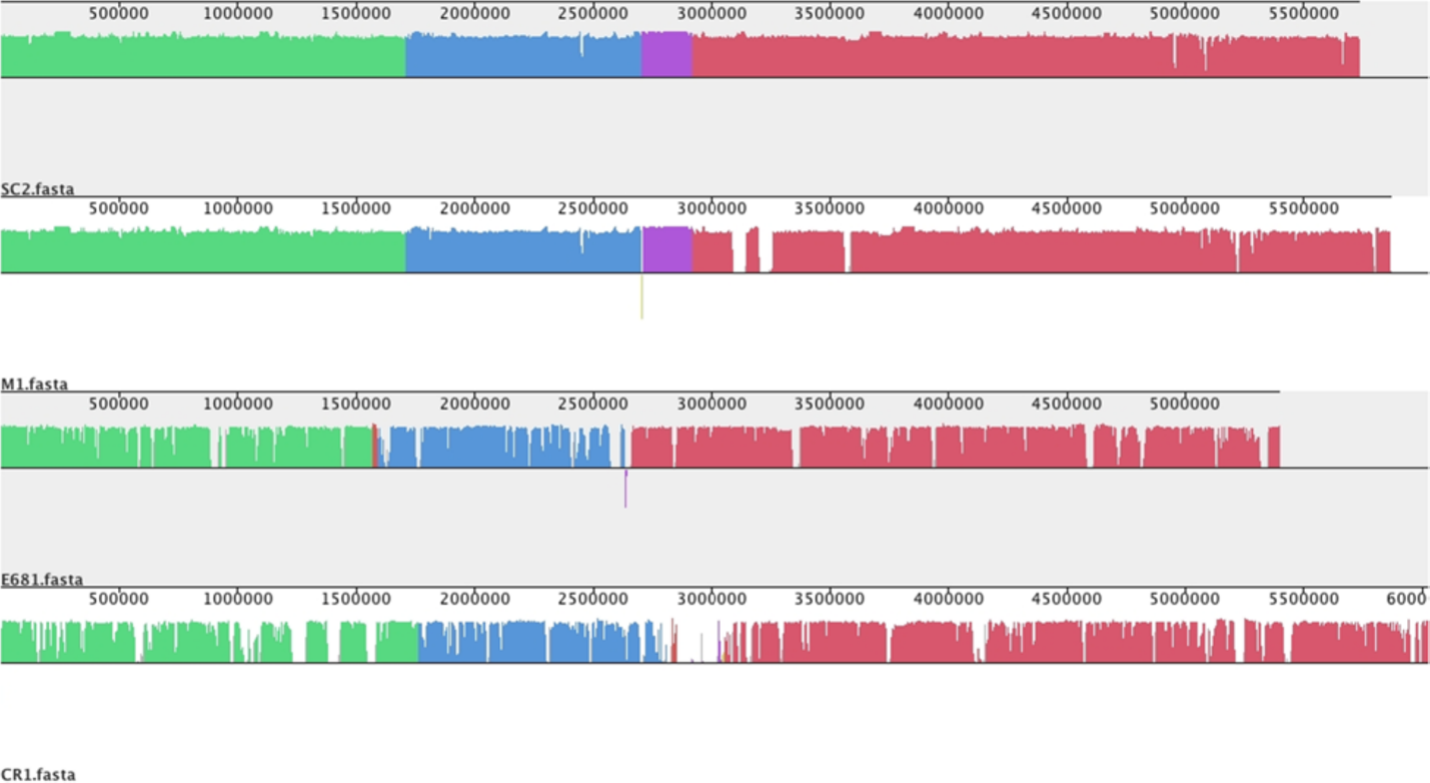
**Global alignment of chromosomes of completely sequenced** ***P. polymyxa*** **strains.** The local collinear block (LCB) plot was generated using the progressiveMauve algorithm using default parameters. The name of the strain represented is listed below each LCB plot. Global alignments are visualized as LCBs, which represent regions of chromosomal similarity among strains. Six LCBs were identified in the four *P. polymyxa* strains genomes and are coloured according to homology to LCBs of other strains. Regions without colour both within and between LCBs represent the presence of strain-specific sequence. LCBs drawn below the horizontal correspond to inversions.

A global alignment of CR1, E681, M1 and SC2 chromosomes was performed using progressiveMauve and visualized as local collinear blocks (LCBs) to glean global information into the nucleotide level similarity amongst the sequenced *P. polymyxa* genomes (Figure 2). The nucleotide level similarity between M1 and SC2 is markedly higher than the similarity between any other grouping of strains (compare LCB composition between M1 and SC2, Figure 2), demonstrating the close relationship of these two strains. Furthermore, the close relationship between the chromosomes of M1 and SC2 versus the more distant CR1 and E681 at the nucleotide level becomes evident, supporting our phylogeny showing the M1 and SC2 strains forming a sub-clade within the *P. polymyxa* species (Figure 1). Also readily apparent is dissimilarity of the CR1 chromosome compared to any other combination of strains, with many strain-specific, low-similarity regions dispersed throughout the CR1 genome (Figure 2), which are discussed later.

### Plasmid encoded genes

**Figure 3:**
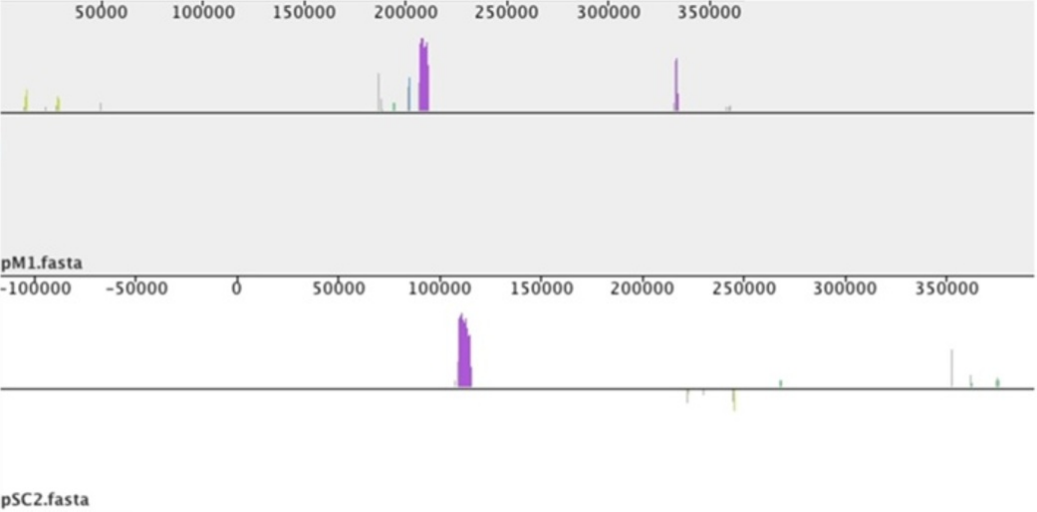
**Global alignment of** ***P. polymyxa*** **plasmids pM1 and pSC2.** The local collinear block (LCB) plot was generated using the progressiveMauve algorithm using default parameters. The plasmid visualized is listed below each respective LCB plot. Global alignments are visualized as LCBs, which represent regions of chromosomal similarity between strains. Five very short and dispersed LCBs were identified between the two plasmids and regions with the same colour represent homologous LCBs in the other plasmid. Regions without colour both within and between local collinear blocks represent presence of strain-specific sequence. Local collinear blocks drawn below the horizontal correspond to inversions. Comparison of pM1 and pSC2 versus their own respective genomes and the genomes of other strains yielded no LCBs and is thus omitted.

Although M1 and SC2 show a high level of similarity (~97% nucleotide identity for the chromosome (Figure 2)), comparison of the M1 and SC2 plasmids at a nucleotide level reveals an absence of homology (Table 2 and Figure 3). In the case of the pM1, 93% of CDS were identified as strain-specific, while 98% of pSC2 CDS were identified as strain-specific. Interestingly, the conserved CDS of pM1 match genes encoded by E681 and CR1, and not genes encoded by pSC2, suggesting the plasmids are unrelated to each other. Notably, pM1 encodes an operon homologous to pyoverdine synthesis genes of fluorescent pseudomonads [48]. Due to the limitations of annotation pipelines, the majority of genes encoded by pSC2 are annotated as hypothetical genes with no conclusive function or experimentally characterized homologs. However, various genes are annotated as metal-dependent hydrolases suggesting SC2 may be dependent on plasmid encoded genes for scavenging nutrients during growth in nutrient-limited conditions. The low homology at the protein and nucleotide levels in conjunction with the large difference in size and number of CDS encoded by pM1 and pSC2, suggests that these two plasmids were recently obtained by an ancestral *P. polymyxa* progenitor strain independently from each other. This notion is further supported when considering the high level of similarity between the core chromosomes of M1 and SC2 (Figure 2). To identify potential ancestral sources of the plasmids, we performed an iterative tBLASTx of the plasmids against the NCBI nucleotide database, which yielded no homology to any known bacterial species or sequenced plasmids (>5% sequence coverage, 70% identity).

### Functional categorization of the *P. polymyxa*genomes

**Figure 4:**
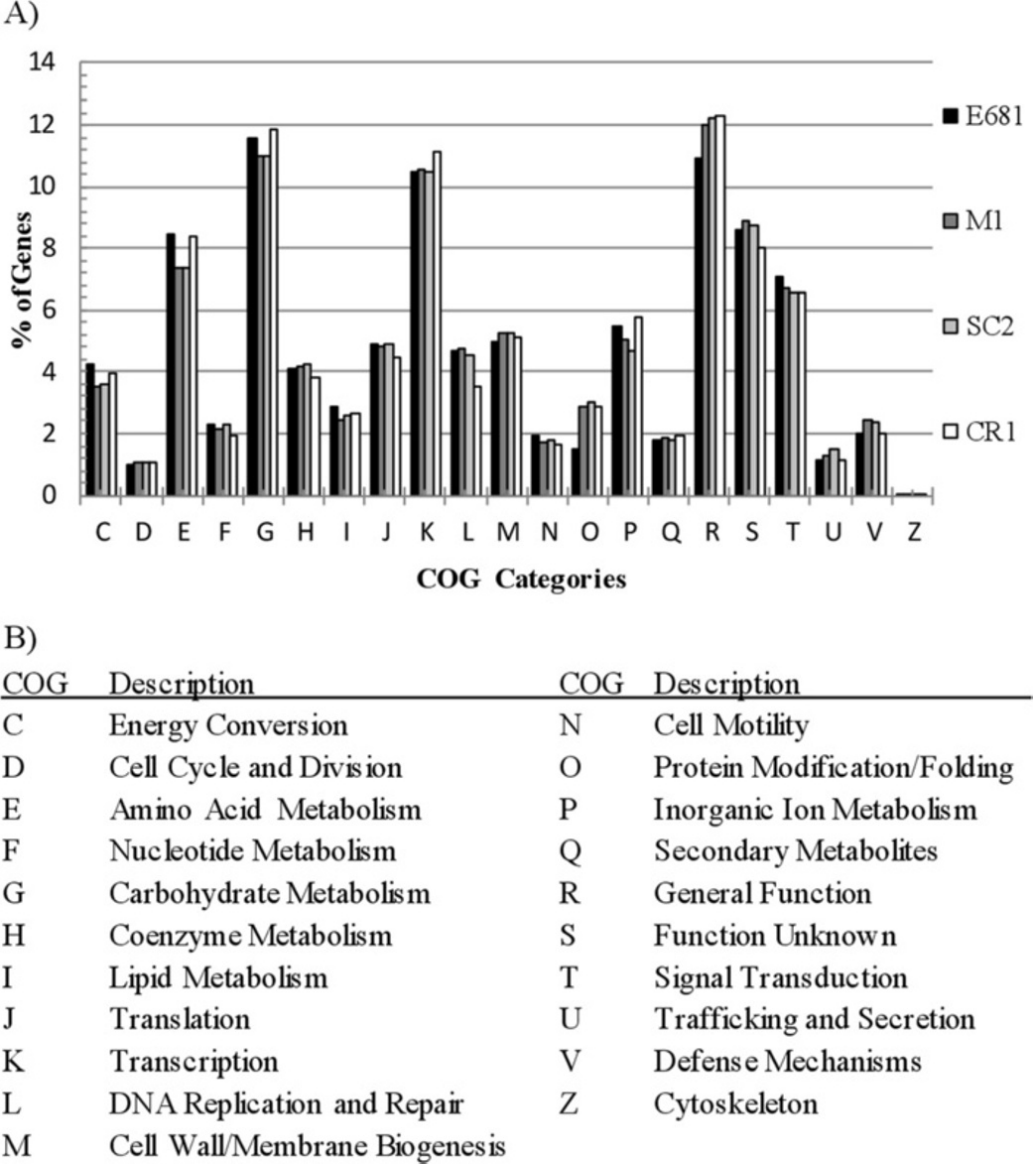
**COG functional categorization of sequenced** ***Paenibacillus polymyxa*** **genomes.** COG functional categorization was performed using available tools on the JGI IMG database. **A)** Proportion of total CDS versus COG categories for the four completely sequenced *P. polymyxa* strains. Functional categories A, B, W, and Y correspond to eukaryotic functions and contained no homologs in any sequenced *Paenibacillus polymyxa* genome and are thus omitted. **B)** List of COG categories and their respective functions.

Comparative analyses of protein function from genomic sequence data of less heavily studied taxonomic families, such as *Paenibacillacae,* are limited by the availability of in-depth experimental data of closely related homologs. The clusters of orthologous groups (COG) categories R and S represent general function prediction and unknown function, respectively, and offer minimal evidence of the substrate specificity and/or protein function of the annotated protein. As evidenced by the large proportion of genes assigned to the COG categories R and S, protein function in the *Paenibacillus* genus is not well characterized. Regardless of the limitations of available data sets and analytical tools, some important inferences into the metabolic capacities of each strain are still possible. The proportion of genes assigned to each COG is presented in Figure 4 to visualize the functional differences between each genome. The COGs A, B, W and Y correspond to eukaryotic functions and thus do not contain any proteins from *P. polymyxa* strains and are omitted [49–51]. Both the CR1 and E681 genomes have a higher proportion of encoded genes dedicated to energy metabolism and inorganic transport and metabolism (categories C, E, G and P), compared to M1 and SC2. The similarity at the nucleotide, protein and functional levels between M1 and SC2 versus E681 and CR1 suggests the two sub-groups diverged and the M1/SC2 ancestor developed into two separate strains after independently obtaining unique plasmids. Conversely, the most recent common ancestor of CR1/E681 likely diverged into each strain earlier giving more opportunities to develop the unique differences unique to each respective strain (Figure 2 and Additional file 1: Figure S1).

### Strain-specific genes

Whole genome alignments of protein coding sequences of the four *P. polymyxa* strains were conducted to visualize the similarity of encoded proteins. Average amino acid identities were calculated using the pair-wise orthologous sets of CDSs from the four *P. polymyxa* genomes. The accessory and core genomes were identified by an *in silico* subtractive hybridization approach using mGenomeSubtractor [52] (Table 1). Homology values (H-values) are defined as an arbitrary unit measuring the degree of similarity between a single protein coding sequence from the reference set and all proteins contained within the user defined subject sets. For purposes of this study we defined the subject sets as all completely sequenced *P. polymyxa* strains and their respective plasmids. H-values <0.41 were considered strain-specific while those >0.81 were considered conserved, representing approximate amino acid level similarity cut-offs of <41% and >81% respectively. When chromosomes and plasmids are considered together all strains contain between 9% and 18% strain-specific CDS. The largest number of strain-specific genes is encoded by CR1 where 955 CDS were identified as strain-specific (18.1% of total CDS), surprising when considering its genome size and absence of a plasmid (Table 1). Interestingly, even with its comparatively small genome size of 5.39 Mb, E681 contained a total of 443 strain-specific CDS. Despite an approximately 10 kb difference in total genome size SC2 contained 625 strain-specific CDS while M1 contained only 394 strain-specific CDS. The chromosomes of strains M1 and SC2 contain very few strain-specific coding sequences, with approximately 2.4% and 0.2% of their chromosome encoded CDS (plasmids excluded) being strain specific on a protein level, respectively (Table 1). Nevertheless, these unique strain-specific CDS may confer some unidentified advantage(s) to the bacterium that allows it to adapt to changing environmental conditions. Further study will be needed to determine the ecological significance of the identified strain-specific genes.

### Horizontally transferred genes

**Figure 5:**
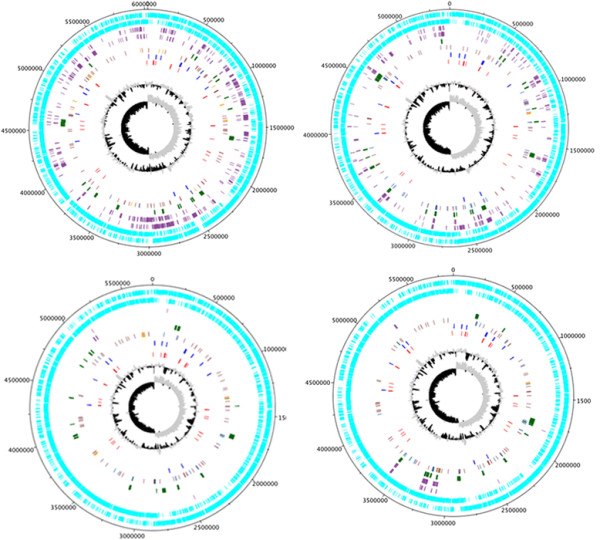
**Circular Representation of the** ***Paenibacillus polymyxa*** **chromosomes.** The rings represent the following features labelled from outside to centre, where the outermost circle represents the scale in Mbps. 1st ring; plus-strand CDS (cyan), 2nd ring; minus-strand CDS (cyan), 3rd ring; plus-strand strain specific CDS (purple), 4th ring; minus-strand strain specific CDS (purple), 5th ring; putative horizontally transferred genes (dark green), 6th ring; phage-related genes (orange), tandem repeats (brick red), transposons (dark blue), 7th ring; ribosomal rRNA genes (bright blue), 8th ring; tRNA genes (red), 9th ring; GC-plot where black and grey correspond to above and below average GC content respectively, 10th ring; GC-skew where black and grey correspond to above and below average GC-skew respectively. Strain-specific genes were identified using mGenomeSubtractor with an H-value cut off of ≤0.41. Putative horizontally transferred genes were identified using IslandViewer 2.0. Annotation was obtained from the NCBI GeneBank database. Phage genes, tandem repeats and transposons were identified using PHAST and IS Finder, respectively. rRNA and tRNA genes were obtained from available annotations.

Foreign DNA acquired by horizontal gene transfer (HGT) is commonly associated with insertion sequence (IS) elements, tRNA genes, tmRNA genes and transposons and is identified by an anomalous GC content, codon bias, di/trinucleotide differences and GC skew [53]. To visualize the general structure of completely sequenced *P. polymyxa* genomes, plus and minus strand CDS, RNA genes, transposons, phage related genes, insertion elements, potential horizontally transferred loci and strain-specific genes were annotated in Artemis [54] and plotted using DNAPlotter [55] (Figure 5). Putative HGT events were identified using IslandViewer 2.0 [56], which scans the target genome for perturbations in the average GC content using a Hidden Markov Model [57]. Although robust, this method omits potential HGT between bacteria with a similar G + C content. Notably, the variation observed earlier among *P. polymyxa* strains (Figure 2) appears to be largely generated through HGT events, as most strain-specific genes correspond to putative HGT loci.

**Figure 6:**
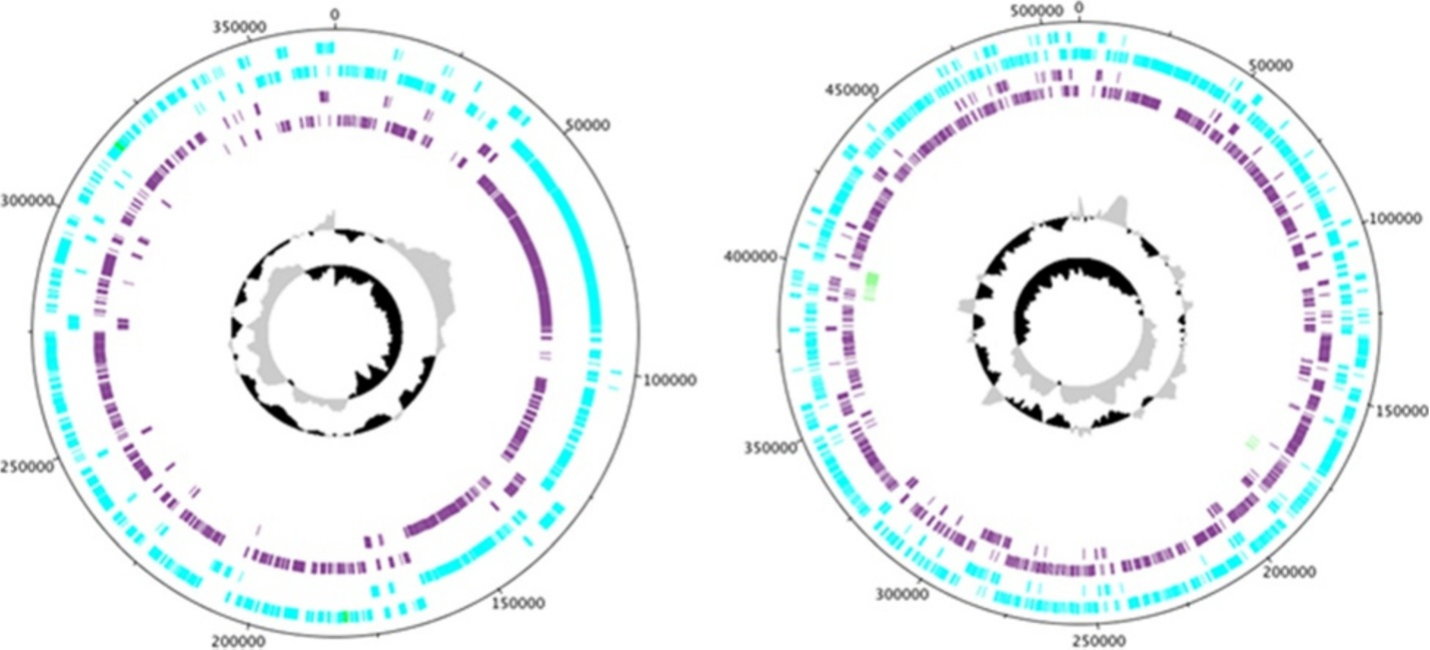
**Representation of** ***Paenibacillus polymyxa*** **strain plasmids.** Plasmids from strains M1 (left) and SC2 (right) were annotated in Artemis and visualized using DNAPlotter. Labelled from outside to centre. Outermost ring shows the scale in Mbps, 1st ring; plus-strand CDS (cyan), 2nd ring; minus-strand CDS (cyan), 3rd ring; plus-strand strain specific CDS (purple), 4th ring; minus-strand strain specific CDS (purple), 5th ring; tRNA genes (green), 6th ring; GC-plot where black and grey correspond to above and below average GC content respectively, 7th ring; GC-skew where black and grey correspond to above and below average skew respectively. Strain specific genes were identified using mGenomeSubtractor with an H-value cut off of 0.42. Annotation was obtained from the NCBI GeneBank database. Phage genes, tandem repeats and transposons were identified using PHAST and IS Finder, respectively. rRNA and tRNA genes were obtained from available annotations.

Genomic islands (GIs) are thought to be genetic elements acquired during evolution from distantly related organisms and such horizontally transferred genes contribute to genome flux and variation [53, 58]. Initially identified and established as important mediators of virulence in pathogenic bacteria, GIs were subsequently identified in non-pathogenic bacteria from niches such as the rhizosphere [53]. Many GIs encode traits that enhance bacterial fitness including iron-uptake systems, polyketide synthesis clusters, resistance cassettes, symbiosis genes, xenobiotic compound degradation and primary metabolism pathways [58]. The locations of identified genomic islands and their encoded genes are provided in Additional file 2. The majority of CDS in the identified genomic islands are annotated as hypothetical genes although many genomic islands include antibiotic synthesis and resistance genes. Some of the most interesting features encoded within identified genomic islands include a bacitracin synthase in E681 and CR1, bacillorin and β-lactamase in SC2, multidrug efflux pumps and sugar hydrolases in M1, as well as a minimal *nif* cluster in CR1. In addition, insertion sequences were identified using IS Finder [58] (Figure 5) and their genome locations are provided in Additional file 3. The *P. polymyxa* strains harbour between 3 and 11 IS elements, and between 1 and 15 transposase containing genomic islands (Figure 5). A large number of transposons, insertion elements, prophages and tandem repeats are contained within genomic islands suggesting *P. polymyxa* is a common phage target. The plasmids pM1 and pSC2 were visualized using the same method described above (Figure 6). Since the majority of genes encoded by the plasmids have unknown functions only plus and minus strand CDS, strain-specific genes and tRNA genes are presented.

### Plant growth promoting traits

**Figure 7:**
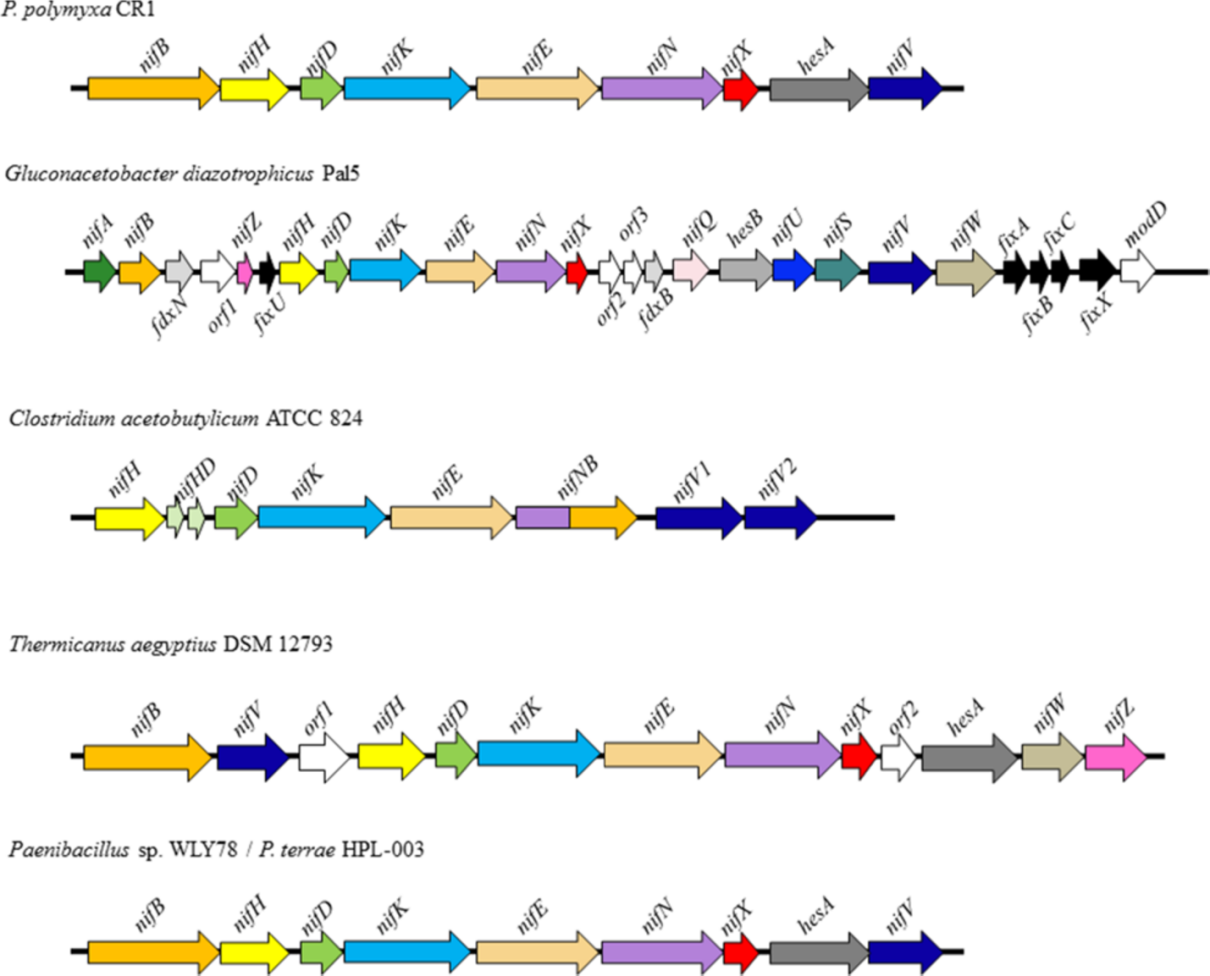
**Comparison of** ***Paenibacillus polymyxa*** **CR1** ***nif*** **cluster to other free-living diazotrophic bacteria.** Genes indicated by the same colour represent functional or structural homologs. Cluster homology is based off of gene clustering using available tools on the JGI Integrated Microbial Genomics Database. Representative *nif* clusters encoded by other free-living diazotrophic bacteria are included for comparison. Bacteria from *Rhizobia* are excluded due to the relative complexity of their nitrogen fixation clusters gene organization, as well as their requirement for nodulation, a trait not observed in *Paenibacillus* sp.

**Figure 8:**
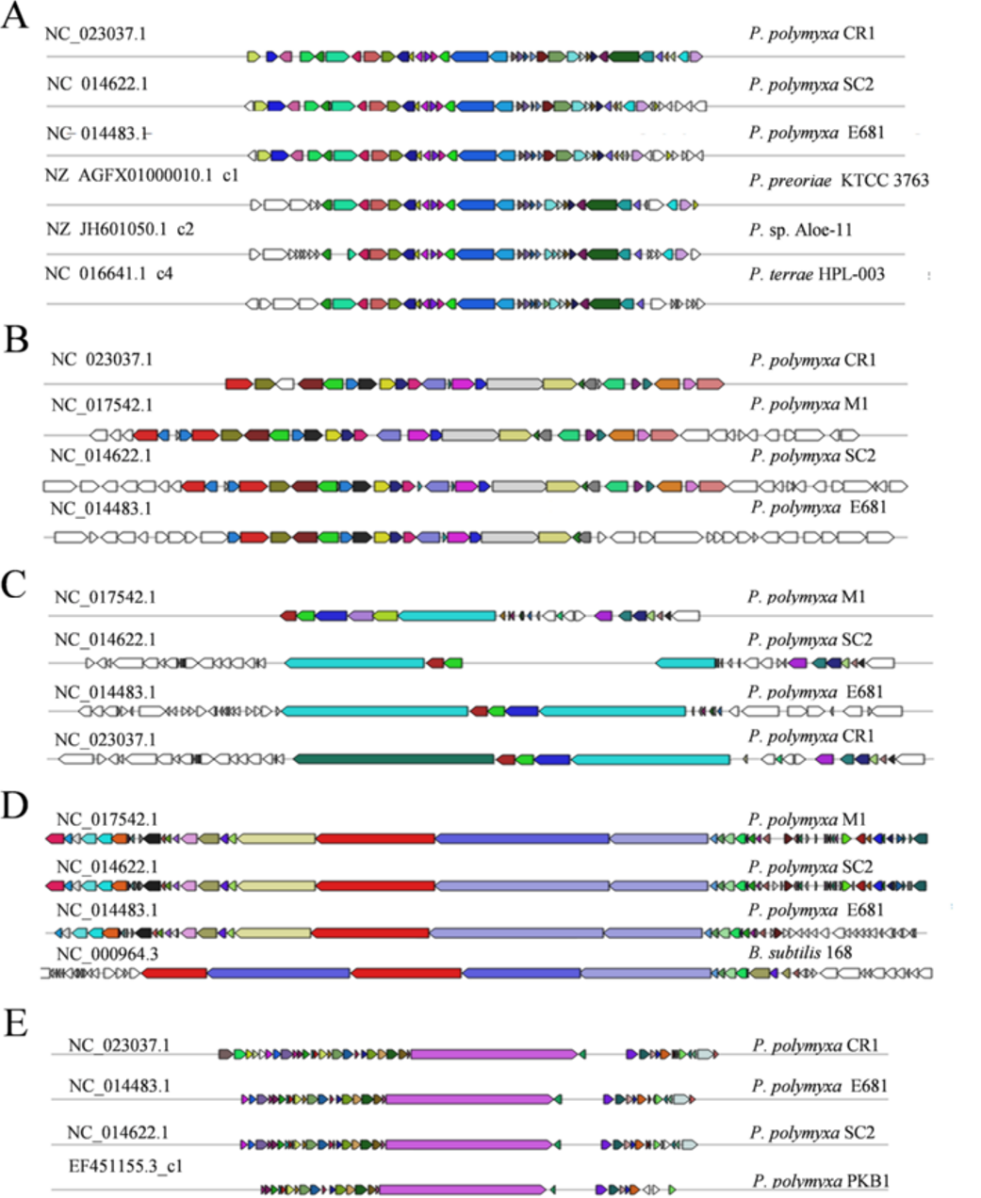
**Non-ribosomal peptide and polyketide synthesis clusters show variation between** ***P. polymyxa*** **strains.** Non-ribosomal peptide synthesis and polyketide synthesis genes were identified using antiSMASH and clustered based off homology to other sequenced bacteria. Only those clusters identified ≥3 *P. polymyxa* genomes are included in the figure. **A)** Bacitracin synthesis cluster. **B)** Lantibiotic synthesis cluster. **C)** Polymyxin synthesis cluster. **D)** Polyketide synthesis cluster. **E)** Fuscaricidin synthesis cluster.

The majority of genes involved in plant-growth promotion and plant-derived compound metabolism were highly conserved amongst *P. polymyxa* strains (Table 3). These include genes responsible for indole-3-acetic acid production, mineral phosphate and phosphonate solubilization, and synthesis of specific antimicrobial non-ribosomal peptides. This is expected as the association of the species with the rhizosphere and endophytic niches are well-documented [9–18]. Notably, presence of genes responsible for nitrogen fixation (*nifB, nifH, nifD, nifK, nifE, nifN, nifX, hesA,* and *nifV,* described in Figure 7), carbohydrate hydrolases (Additional file 4: Table S1) and bio-control compounds (Figure 8) varied between strains. These traits have potential applications for sustainable agricultural and environmental processes aimed at reducing dependency on chemical fertilizers and pesticides, which are discussed in the follow sections.

**Table 3:** **Plant Growth Promoting Genes of** ***P. polymyxa*** **strains**

Trait	***Gene Name***	CR1	E681	M1	SC2
Indole-3-acetic acid production					
	*ipdC*	YP_008911027	YP_003869749	YP_005958986	YP_003945692
	auxin efflux carriers	YP_008912813 YP_008912323 YP_008911849	YP_003871347 YP_003870860 YP_003870585	YP_005960839 YP_005960204 YP_005959850	YP_003947565 YP_003947053 YP_003946661
Phosphate solubilization					
	*gcd*	YP_008912273	YP_003870830	YP_005960174	YP_005960174
Phosphonate cluster (*phn*)					
	*phnA*	YP_008914717	YP_003873107	YP_008050528	YP_003949521
	*phnB*	YP_008910326	YP_003869234	YP_003945144	YP_005958491
	*phnC*	YP_008913947	YP_003872434	YP_008049904	YP_003948836
	*phnD*	YP_008913946	YP_003872433	YP_008049903	YP_003948835
	*phnE*	YP_008913948	YP_003872435	YP_008049905	YP_003948837
	*phnW*	YP_008914692	YP_003873086	-	-
	*phnX*	YP_008909947	YP_003868868	YP_005958118	YP_003944734
	*ppd*	YP_008914693	YP_003873087	-	-
	*pepM*	YP_008914694	YP_003873088	-	-
Phosphate transporter (*pst*)					
	*pstS*	YP_008911198	YP_003869955	YP_005959210	YP_003945962
	*pstA*	YP_008911200	YP_003869957	YP_005959212	YP_003945964
	*pstB*	YP_008911201	YP_003869958	YP_005959213	YP_003945965
	*pstC*	YP_008911199	YP_003869956	YP_005959211	YP_003945963
	*phoP*	YP_008911212	YP_003869969	YP_005959224	YP_003945977
	*phoR*	YP_008911211	YP_003869968	YP_005959223	YP_003945976
Nitrogen fixation					
	*nifB*	YP_008910495	-	-	-
	*nifH*	YP_008910496	-	-	-
	*nifD*	YP_008910497	-	-	-
	*nifK*	YP_008910498	-	-	-
	*nifE*	YP_008910499	-	-	-
	*nifN*	YP_008910500	-	-	-
	*nifX*	YP_008910501	-	-	-
	*hesA*	YP_008910502	-	-	-
	*nifV*	YP_008910503	-	-	-

"-" corresponds to no genes with homology to the gene listed on the left. Accession numbers listed refers to protein sequences in the NCBI Protein database. Genes were identified using annotations provided in Genebank followed by BLASTx searches of the genomes using previously characterized homologs. Auxin efflux carrier proteins were identified using Transporter Classification on the JGI IMG database.

### Biological nitrogen fixation

Nitrogen is a critical limiting element for plant growth and production in agricultural systems, since plants are only capable of incorporating reduced forms of nitrogen (ammonia, nitrates etc.) [59]. Nitrogenase, an oxygen sensitive dinitrogen reductase encoded by certain microorganisms converts inert atmospheric nitrogen (N_2_) into ammonium (NH_4_) thereby improving plant growth and crop yields by increasing the concentration of biologically available nitrogen [60]. Recent research on *Paenibacillus sp.* WLY78 has demonstrated that a minimal *nif* cluster can confer nitrogen fixation to *Escherichia coli* [61]. In this study, a minimal *nif* cluster was identified only in CR1 but not the other three *P. polymyxa* genomes. The gene organization and composition of the CR1 *nif* cluster was identical to the previously identified *nif* cluster of WLY78 [61]. The entire *nif* cluster of CR1 is approximately 10.5 kb and encodes 9 genes; *nifB, nifH, nifD, nifK, nifE, nifN, nifX, hesA,* and *nifV* (Figure 7)*.* The *nifHDK* genes encode a Mo-nitrogenase, the enzyme responsible for fixing-nitrogen, while *nifBENX* and *nifV* are responsible for the synthesis and maturation of the FeMo-cofactor. The gene *hesA* encodes a NAD/FAD-binding protein involved in molybdopterin and thiamine biosynthesis. Interestingly, the identified *nif* gene cluster has higher G + C mol% content (53.4%) than the rest of *P. polymyxa* genome (45%) and the *nif* cluster was identified as a GI by IslandViewer (Additional file 2), suggesting this *nif* cluster may originate from a distantly-related higher G + C content bacterial species. However, comparison of the minimal *nif* cluster of CR1 with available genomic data on the Joint Genome Institute Integrated Microbial Genomics Database [62] and NCBI Genebank databases did not reveal a potential source of the *nif* cluster. However, an identical *nif* cluster (Figure 7) was also identified in *Paenibacillus terrae* HPL-003 [63], the next closest related *Paenibacillacae* to the *P. polymyxa* species (Figure 1). Therefore, we speculate that *P. polymyxa* and *P. terrae* are likely derived from the same ancestor possessing the *nif* cluster, which would explain the identical cluster organization and high nucleotide level similarity between the CR1 and HPL-003 *nif* clusters (94% identity, E-value 0.0). The whole genome phylogeny (Figure 1) taken in concert with the presence of the *nif* cluster in *P. terrae* HPL-003 (Figure 7) suggests that the ancestor of *P. polymyxa* strains encoded the *nif* cluster which was subsequently lost in the E681/M1/SC2 strains.

Unlike free-living Gram-negative nitrogen fixing bacteria such as *Gluconacetobacter diazotrophicus* [64, 65], there is no *nifA* gene in *Paenibacillus* sp. or other Gram-positive, diazotrophs such as *Clostridium* sp. The *nifA* gene encodes a *nif*-specific regulatory protein that activates the expression of *nifHDK* genes [64]. In the N_2_-fixing alfalfa symbiont *Sinorhizobium meliloti*, the three sigma-54 type NtrA-dependent regulatory proteins NifA, NtrC, and DctD are required for activation of promoters involved in N_2_-fixation (*nif* genes), nitrogen assimilation (*pgln*II), and C4-dicarboxylate transport (*pdctA*) respectively [66, 67]. Previous experiments revealed that the *nifA* gene is dispensable for N_2_-fixation since a mutation in *ntrC283* allele in *S. meliloti* restores the N_2_-fixation ability of a *nifA* deletion mutant to 70-80% of the wild-type level [68, 69]. Therefore, it is possible that the NtrC homolog encoded by CR1 (YP_008912290.1) activates expression of the *nif* gene cluster. The other three *P. polymyxa* strains do not contain the NtrC homolog found in CR1, consistent with the absence of the *nif* gene cluster in these three strains. However, it is unclear how these three *P. polymyxa* strains (E681, M1 and SC2) handle nitrogen assimilation.

In *Rhizobia*, DctD, a C4-dicarboxylate response regulator, activates the expression of *dctA* upon detection of C4-dicarboxylates. Previous studies have found that DctD is not only involved in C4 uptake, but also participates in symbiotic nitrogen fixation through complex signalling mechanisms [69, 70]. Interestingly, our analysis also revealed a *dctD* homolog in the CR1 genome, which matched to the same protein identified as an NtrC homolog (YP_008912290.1). These results suggest that CR1 maintained the *nif* cluster and a hybrid NtrC/DctD response regulator required for nitrogen fixation. Conversely the absence of *ntrC* and *dctD* gene homologs in the other *P. polymyxa* strains is unsurprising since these strains lack the *nif* cluster and do not require their associated signalling pathways. In the case of a recent study that demonstrated *Escherichia coli* nitrogen fixation by acquisition of the *nif* cluster from *Paenibacillus sp.* WLY78 [61], it is plausible that the NtrC homolog in *E. coli* partially activates expression of the exogenous *nif* cluster. Nevertheless, the 9 gene *nif* cluster identified in CR1 is particularly exciting for its potential use as a transferable nitrogen fixation genetic element to facilitate development of genetically modified plant-growth promoting bacteria. The broad host-range and free-living lifestyle of *P. polymyxa* coupled with the ability to fix nitrogen is in stark contrast to *Rhizobia* sp. which require nodule organogenesis (mediated by *nod* genes) prior to induction of diazotrophy [71].

### IAA production

A major mechanism employed by PGPR to enhance plant growth is the production of various plant hormones including auxins, gibberellins, and cytokinins, or other factors that modulate plant regulatory systems [2, 3, 7, 8, 72–74]. Indole-3-acetic acid (IAA) is the primary auxin endogenously synthesized by plant-associated bacteria and has a profound effect on plant growth and development. Auxins produced by plant-associated bacteria typically induce root branching and elongation in the plant hosts, thereby enhancing plant fitness/growth, and thus increasing available plant derived nutrients for the bacteria in return. We compared the sequenced *P. polymyxa* genomes for pathways involved in the production of IAA. Indole-3-pyruvate decarboxylase (IPDC) is a key enzyme involved in the IPA pathway and is encoded by the *ipdC* gene, which has been established as necessary for the production of IAA in E681 [31]. Highly similar genes (≥97% amino acid identity, ≥98% coverage) are present in M1, SC2 and CR1 (Table 3). However, genes encoding tryptophan-2-monooxygenase and indole-3-acetamide hydrolase, which convert tryptophan to IAA via the two-step indole-3-acetamide pathway, were not detected in the four genomes. This suggests the indole-3-pyurvatic acid (IPA) pathway is the sole IAA production mechanism employed by *P. polymyxa*. Preliminary results in our lab corroborated the capacity to produce IAA by the IPA pathway in CR1 (Weselowski *et al.*, manuscript in preparation). Furthermore, all genomes examined here encode putative auxin efflux carrier proteins, suggesting that these bacteria are capable of production and export of IAA in a tryptophan-dependent manner (Additional file 5). However, our data suggests CR1 may also be able to produce IAA in a tryptophan-independent manner, which has yet to be conclusively demonstrated. Since CR1 is a tryptophan prototroph, further experiments are needed to control for IAA production from endogenous tryptophan obtained either through *de novo* synthesis or protein catabolism. Research is inconclusive on the production of cytokinins and gibberellins in *P. polymyxa* and if the produced amounts have a physiological effect on plants [72]. To the best of our knowledge no characterization of genes responsible for cytokinin and gibberellin production has been performed in any closely related species.

Aminocyclopropane-1-carboxylic acid (ACC) is the immediate precursor of the plant hormone ethylene. Stressed plants accumulate ethylene, which inhibits root elongation and accelerates abscission, aging and senescence. ACC deaminase-producing rhizobacteria lower plant ethylene levels by converting ACC into ammonia and α-ketobutyrate, preventing inhibition of root growth thereby improving tolerance to environmental or pathogen-induced stress [5, 8]. The *acdS* gene encodes ACC deaminase, however no *acdS* homologs were identified amongst the four sequenced strains.

### Phosphate solubilization and assimilation

Phosphate is a major limiting nutrient in soils and a large proportion of phosphate in soils is sequestered in mineral compounds [5, 75]. Solubilization of mineral phosphates by bacteria is typically achieved through gluconic acid production. Previous research in various *Pseudomonas* sp. have implicated glucose-1-dehydrogenase *(gcd)* and gluconic acid dehydrogenase (*gad)* in the production of gluconic acid and its conversion to 2-ketogluconate respectively [76]. The lowered pH resulting from the secretion of gluconic acid results in formation of phosphorylated gluconate, which in turn is up taken by sugar-specific transporters. Our characterization demonstrated CR1 is capable of solubilizing inorganic mineral phosphates thereby increasing phosphate availability for the plant host (unpublished data). In this study, we identified putative genes encoding glucose-1-dehydrogenase and gluconic acid dehydrogenase in all sequenced *P. polymyxa* genomes (Table 3), suggesting that phosphate solubilization is likely mediated through gluconic acid secretion, and that all strains are capable of solubilizing mineral phosphates.

Another rich source of soil phosphate is trapped in the form of phosphonate, an organophosphorus compound that must be degraded prior to biological incorporation. The phosphonate gene cluster (*phn*) is responsible for bacterial degradation of phosphonates, which releases biologically available phosphate for nearby plants. Our comparative genomics revealed that *P. polymyxa* strains do not carry the complete *phn* cluster, lacking the genes encoding a C-P lyase protein (*phnGHIJKLM)* responsible for phosphonate degradation into phosphate and an alkane, a system commonly identified in other phosphonate degrading bacteria (Table 3). All *P. polymyxa* strains appear to possess the capability to degrade phosphonoacetaldehyde and phosphonoacetate (*phnX, phnA*). In addition, CR1 and E681 also possess the necessary genes *(ppd, pepM)* for 2-aminoethylphosphonate and phosphonopyruvate degradation. Furthermore, CR1 and E681 encode *phnW,* required for the biosynthesis of phosphonates, possibly utilizing produced phosphonates as a sequestered storage form of phosphate (Table 3).

### Antimicrobial compound production

Bacteria often produce antimicrobial peptides and proteins called bacteriocins to suppress surrounding bacteria to gain a colonization advantage over competing bacteria. Enzymes responsible for synthesis of non-ribosomal peptides (NRP) and polyketides (PK) are modular in nature and are comprised of a multitude of possible modification domains including adenylation, condensation, thiolation and esterification domains. The most well-known NRPs produced by *P. polymyxa* are polymyxin B/E, which are used in over-the-counter antimicrobial ointments and renewed applications as a last resort antibiotic against multiple drug resistant bacteria [77]. There is evidence *P. polymyxa* co-produces NRPs, presumably as an aggressive defense response against potentially hostile bacteria [78]. Recent research has shown that antimicrobial compounds produced by *Paenibacillus* sp. target a variety of both pathogenic and non-pathogenic bacteria and fungi [6, 9, 21, 23–25, 79].

Our analyses identified a large number of heterogeneous NRP, PK and hybrid NRP-PK synthase modules in the genomes of *P. polymyxa* strains, which are summarized in Figure 8 (genomic loci are provided in Additional file 6). However, all *P. polymyxa* strains do not possess identical clusters of NRP and PK synthesis genes, likely representing adaptations by strains to their specific ecological niche and the organisms it competes with. Notably, each strain has an impressive repertoire of strain specific NRP, PK and NRP-PK hybrid synthases. For example, CR1 encodes a staphyloferrin-like-siderophore synthetase, tyrocidine synthetase, a type II polyketide synthase, a type I polyketide synthase and a novel NRP-PK hybrid synthase not found in other bacteria. Meanwhile, E681 encodes a novel gramicidin-like synthase, and a NRP-PK hybrid synthase also found in SC2. M1 encodes fuscaricidin synthase, two NRPs, lichenysin and a lantibiotic otherwise found only in SC2 as well as a plasmid encoded NRP and pyroverdine siderophore. Finally, SC2 encodes gramicidin, two NRPs and a lantibiotic otherwise found only in M1 and a NRP-PK hybrid also found in E681 (homology of NRPs and PKs contained in three or more strains are visualized in Figure 8).

### Iron acquisition

Iron acts as a co-factor and electron acceptor in various essential enzymes and proteins and is an important nutrient to organisms. Siderophores are low molecular weight, ferric ion-specific chelating agents, synthesized by microorganisms growing in iron limited environments [33]. Plant-growth promoting bacteria use NRP synthases to produce siderophores responsible for scavenging heme and non-heme iron from the rhizosphere, increasing its availability for associated plant hosts [5]. Once iron is depleted in the surrounding environment, other microorganisms that do not produce siderophores cannot obtain necessary iron and are growth inhibited. Therefore, siderophore production can act as an antagonistic mechanism by scavenging scarce iron from the soil environment. Our comparative analysis revealed that only CR1 and M1 encode siderophore synthesis clusters. The siderophore synthesis cluster of CR1 shows homology to a siderophore synthesis cluster of *Staphylococcus aureus*, a heavily studied human pathogen. Interestingly, the siderophore synthesis genes are encoded by M1 on its plasmid and show homology to pyoverdine synthesis genes of fluorescent *Pseudomonads,* suggesting siderophore synthesis genes in *P. polymyxa* are obtained by horizontal gene transfer events. Our comparative genomic analyses reveals *P. polymyxa* strains SC2 and E681 do not encode any canonical siderophore NRP synthetase clusters. Therefore, E681 and SC2 either cannot accumulate iron or obtain iron through yet unidentified mechanisms.

### Biomass degradation and bioproduct formation

Bacteria that form commensal or mutualistic relationships with plants are at a selective advantage if they can metabolize plant-derived carbon sources, which are enriched in the rhizosphere environment [5, 80]. Therefore, understanding the bacterial metabolic network is crucial in understanding complex plant-bacterium interactions. The complexity of the *P. polymyxa* metabolic network is reflected by the ability to metabolize lignin or cellulose as a sole carbon source, which aids survival in diverse environments and allows for colonization of a variety of plant species. Recent biofuel technologies are limited by the availability of highly active hydrolytic enzymes that mediate the degradation of complex plant polymers [81]. Our initial characterization revealed CR1 is capable of growth using various plant-derived carbon sources including lignin, cellulose and hemi-celluloses (Weselowski *et al.,* manuscript in preparation). Since highly active hydrolytic enzymes and bacterial strains are a desirable commodity in the developing biofuel sector we compared the quantity and nature of glycoside hydrolases (GH) amongst *P. polymyxa* strains to glean an overall outlook of the strain-specific biomass degradation capacity (Additional file 4 Table S1). We found all strains of completely sequenced *P. polymyxa* encode a large number of various GH family proteins with CR1 encoding the largest repertoire of GH family proteins (133 GH family proteins), followed by M1 (123 GH family proteins). CR1 encodes an increased quantity of GH families 1, 2, 3 and 42 proteins (xylanase and endo/exo-glucanase enzymes) while showing a decreased number of GH family 5 (cellulase) proteins. As a result of their increased number of cellulase domain containing enzymes, M1, E681 and SC2 are also likely proficient cellulose degraders. Interestingly, E681 encodes a large number of rhamnogalacturonan lyase and pectin lyase proteins, which are responsible for non-hydrolytic cleavage of pectin, possibly underpinning a previously unknown saprophytic niche occupied by E681 in soils. Carbohydrate hydrolase enzymes function as complex, multi-meric proteins assembled from various numbers of encoded domains. This allows the substrate specificity of the complex to vary greatly dependant on the stoichiometry of the subunits [82]. It is therefore difficult to make conclusive arguments as to the degradation potential of each strain merely based on a bioinformatics approach. Regardless, the large arsenal of GH domain enzymes makes *P. polymyxa* an untapped reservoir of potentially valuable hydrolytic enzymes and plant biomass degradation complexes. Furthermore, the large quantity of GH family genes present in *P. polymyxa* genomes is consistent with the intimate association of *P. polymyxa* with plant hosts in the rhizosphere where there is an increased concentration of plant-derived compounds.

Lignin metabolism by many actinomyces bacteria as well as various fungi occurs through non-specific redox and free-radical generating mechanisms. Fungal laccase enzymes are copper-dependent hydrolases expressed by white and brown rot fungi that non-selectively depolymerize lignin through reduction-oxidation reactions [83–85]. Conversely, bacterial encoded DyP-family peroxidases have been shown to be capable of degrading phenolic dyes, whose structures mimic complex lignin polymers. Notably, the genomes of all sequenced *P. polymyxa* contain putative DyP-peroxidase and laccase genes (Table 4). Recent research has suggested there are two subfamilies of DyP-peroxidase genes, the A subtype, capable of liberating iron from heme, while the B subtype possesses lignin degrading capacities. Analyses of the putative DyP-peroxidases encoded by *P. polymyxa* using hhPRED [86] and PHYRE 2 [87] suggests structural homology to the DyP type B peroxidase of *Rhodococcus jostii* RHA1 (accession no. 3QNR_A) that is responsible for non-specifically degrading lignin substrates [88, 89]. Furthermore, our preliminary data demonstrates CR1 decolours extracellular methylene and toluidine blue dyes, suggesting the DyP peroxidase is secreted and may be responsible for the lignin degrading phenotype. Further experimental data will be needed to determine their respective functions and their applicability to industrial processes.

**Table 4:** **Putative ligninolytic enzymes of sequenced** ***Paenibacillus polymyxa*** **strains**

Gene homology	Accession number			
	CR1	E681	M1	SC2
DyP type peroxidase	YP_008913589	YP_003872107	YP_008049549	YP_003948444
Laccase	YP_008912908	YP_003871475	YP_008048909	YP_003947734

Location refers to the chromosome location on the genome of each respective strain. Accession numbers refer to the protein sequence contained in the NCBI Protein database. Genes encoding putative Dyp type peroxidases were identified based off of BLASTx homology with DyPB from *Rhodococcus jostii* RHA1. Laccase enzymes were based off of annotations provided by the NCBI Genebank database and homology to fungal encoded laccase enzymes.

**Figure 9:**
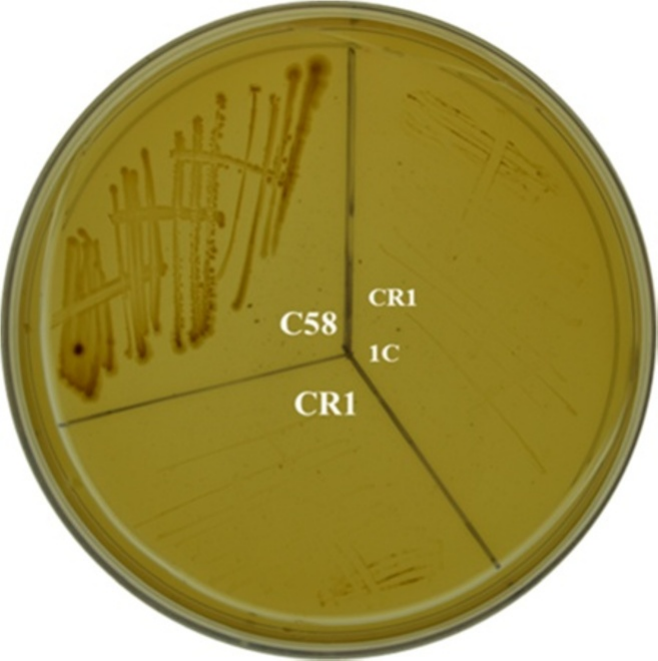
***P. polymyxa*** **CR1 cannot utilize protocatechuate as sole carbon source.** *P. polymyxa* CR1 and its derivative strain 1C were grown on minimal media with protocatechuate as the sole carbon source for 4 days at 37°C. *Agrobacterium fabrum* C58 protocatechuate degradation have been established previously and acts as a positive control.

Specific strains of *P. polymyxa* are noted for their ability to sterioselectively ferment valuable (R, R)-2, 3-butanediol from monosaccharide feed stocks [90–92]. Preliminary data from our lab shows CR1 is capable of fermenting Kraft lignin into a mixture of methylated and non-methylated short chain alcohol compounds (unpublished data). Our attempts to characterize the lignin-degrading pathway by a computational approach are greatly limited by the lack of detailed studies of *Firmicutes* lignin-degrading pathways. Further studies are necessary for elucidating the responsible enzymes and regulatory networks. Further complicating our analysis, our research suggests CR1 is incapable of degrading protocatechuate (Figure 9), a key nodal metabolite in currently characterized Gram-negative lignin degradation pathways [89]. This is consistent with the lack of identified homologs for the protocatechuate cleavage pathway in all sequenced *P. polymyxa* strains, as determined using KO classification and BLASTx searches of characterized genes (*pca* genes) from characterized species. Therefore, *P. polymyxa* likely utilizes a yet to be characterized lignin and aromatic degradation pathway and to the best of our knowledge no complete characterization of the lignin metabolism network has taken place in any *Firmicutes* bacteria.

### Regulatory network and stress response

In variable and changing environments bacteria with larger genomes encoding varied metabolic capabilities have a long-term selective advantage [43, 93]. The soil environment is heterogeneous with an irregular distribution of a variety of substrates, nutrients, antagonistic compounds and potential stressors. Accordingly, many soil-dwelling, plant-associated bacteria have relatively large genomes, presumably to allow the bacterium to survive in such a variable environment [94]. Soil bacteria coordinate multicellular behavioural responses to integrate environmental cues through complex regulatory networks. Consistent with this premise, *P. polymyxa* encodes a large number of transporters and regulators allowing a wide range of substrates to be up taken. Bacterial two-component signal transduction systems play important roles in enabling detection and response to diverse changes/stresses in the environment [95, 96]. The *B. subtilis* DegS/DegU two component system is involved in the regulation of post-exponential phase processes, including activation of genetic competence, motility, poly-γ-glutamic acid production and biofilm formation [97, 98]. During the transition from exponential to stationary growth, *B. subtilis* produces several proteolytic and hydrolytic enzymes including an intracellular protease and several secreted enzymes (levansucrase, alkaline and metalloproteases, a-amylase, 3-glucanase(s), and xylanase), which are controlled at the transcriptional level by the DegS/DegU two-component system. To understand how *P. polymyxa* co-ordinates the decision-making processes to ensure appropriate physiological responses occur during different environmental conditions, we attempted to identify a *P. polymyxa degS*/*degU* two-component system. Our analysis at the genome level indicates that a conserved *degS* (CR1: YP_008914314.1; M1: YP_008050147.1; SC2: YP_003949105.1; E681: YP_003872721.1) and *degU* (CR1: YP_008914313.1; M1: YP_008050146.1; SC2: YP_003949104.1; E681: YP_003872720.1) are present among the sequenced *P. polymyxa* genomes (E-value <10^−75^, >70% positive amino acid identity). However, without experimental evidence, we can only speculate the identified two-component system DegS/DegU participates in biomass degradation and provides a selective advantage to *P. polymyxa*.

Motility is a fundamental process employed by many bacteria during adaptation to environmental changes and stresses. Chemotactic pathways governed by two-component regulatory systems in response to specific signals are mediated through methyl-accepting chemotaxis proteins (MCPs). The repertoire of chemotactic signal pathways of a microorganism is correlated to the environments it inhabits and the hosts it colonizes [95]. Many *Bacilli,* including *P. polymyxa*, also encode anti-sigma-factors (anti-σ-factors) or anti-anti-σ-factors that are secreted either as a mechanism of competition with other bacteria or to control their own gene expression in rapidly changing environments where tight gene regulation is paramount. Strikingly, all strains of *P. polymyxa* encode a large number of σ-factors and MCPs (Additional file 4: Table S2). In particular, all *P. polymyxa* strains contain a full complement of σ-factors related to spore formation, corroborating our experimental evidence of the propensity of CR1 to form spores in nutrient limited conditions (unpublished data). As expected, *rpoD*, *sigE*, *rpoN* and *fliA* are encoded by all strains, representing σ-factors responsible for basal transcription, general stress responses, nitrogen limitation and flagella assembly respectively. In addition to necessary basal transcription factors, each strain encodes a variable amount anti-σ-factors and anti-anti-σ-factors, which may be involved in sensing environmental conditions and modulating gene expression accordingly (Additional file 4: Table S2). M1 encodes 22 extracellular σ-factors, slightly more than SC2, which encodes 20. CR1 encodes 15 extracellular σ-factors, while E681 encodes the fewest with only 13 extracellular σ-factors. Unexpectedly, E681 encodes 27 MCPs despite having the smallest genome and fewest σ-factors, suggesting E681 prefers to migrate to higher nutrient environments as opposed to tight genetic control in nutrient limited conditions. Both SC2 and M1 encode 25 MCPs molecules while CR1 encodes only 24. The abundance of predicted MCPs and σ-factors indicates an elaborate sensing capability of *P. polymyxa* that presumably allows the bacterium to survive in different ecological niches.

### Quorum sensing

**Figure 10:**
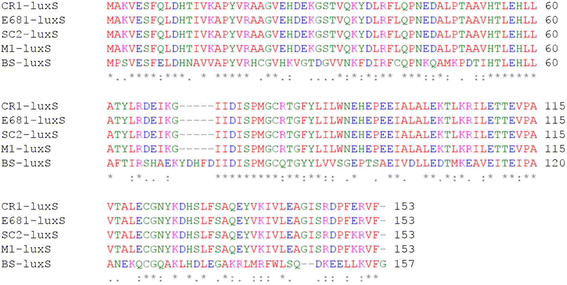
**ClustalΩ alignment of LuxS homologs encoded by** ***P. polymyxa*** **strains.** Putative LuxS homologs were identified based off BLASTp searches using the characterized *Bacillus subtilis* LuxS protein. Amino acid alignments were performed using ClustalΩ and default parameters.

Quorum sensing (QS) is a specialized cell-to-cell communication mechanism employed by bacteria to control gene expression on a community level through secretion and detection of extracellular signalling molecules, called autoinducers, which accumulate in the environment proportional to cellular density [99]. QS-controlled behaviours include bioluminescence, biofilm formation, virulence, antimicrobial compound production and competence [83–86]. The complex role of quorum sensing in the *P. polymyxa* lifestyle has not yet been revealed so far, however, other members of the genus are known for intricate signalling pathways and striking patterns formed during migration [100]. The ComQXPA system of *B. subtilis* is a typical Gram-positive QS system that controls bacterial competence [97, 101]. However, our genomic analysis reveals no homologs of *comQXPA* genes in the *P. polymyxa* genomes, which may explain the low transformation frequency of CR1 compared to *B. subtilis* (our unpublished data). In addition to ComQXPA, *B. subtilis* operates a LuxS-dependent QS system that regulates its morphogenesis and social behaviour [102, 103]. The *luxS* gene product synthesizes autoinducer-2 (AI-2) that modulates biofilm formation. Expression of *luxS* is negatively regulated by two master regulatory proteins, SinR and Spo0A. SinR is a key regulator for proper biofilm development and Spo0A controls sporulation in *B. subtilis* [100, 101]. Our comparative genomics revealed a conserved *luxS* gene homolog among the fully sequenced *P. polymyxa* genomes (Figure 10). In *Vibrio harveyi*, two proteins, LuxP and LuxQ, function together as the AI-2 sensor. LuxP is a periplasmic binding protein, and LuxQ is a hybrid two-component protein that contains both a sensor kinase and response regulator domain [104]. However, genomic analyses did not identify a *luxP* homolog among these sequenced *P polymyxa* genomes, instead, a conserved *luxQ* homolog was found within the *P. polymyxa* genomes (CR1: YP_008913839.1, M1: YP_008049796.1; E681: YP_003872339.1; SC2: YP_003948716.1). Therefore, it is plausible that in these *P. polymyxa* strains, LuxS is the main QS signal producer, whereas, the hybrid two-component protein LuxQ plays a major role as a sensor kinase and response regulator for the LuxS-dependent QS amongst strains of the species.

### Transport mechanisms

**Figure 11:**
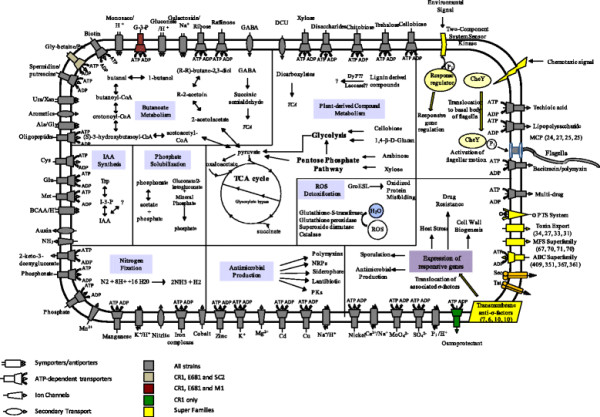
**Schematic overview of** ***Paenibacillus polymyxa*** **metabolism.** Listed beside each superfamily is the number of CDS found in the following order; CR1, E681, M1, SC2. Metabolic and regulatory pathways involved in survival in the rhizosphere niche and plant-growth promoting traits are included in the interior of the cell diagram.

Soil bacteria are known to be rich in ATP-binding cassette (ABC) transporters. *P. polymyxa* is typically found either in association with plant hosts or as free-living in the rhizosphere and is expected to encode diverse metabolic networks and transporters to adapt to the specialized living environments [81]. To gain insights into the potential metabolic pathways we compared encoded transporter families and their specificities amongst sequenced *P. polymyxa* genomes (Additional file 4: Table S3 and summarized in Figure 11). CR1 encodes both a larger absolute number and proportion of ABC-transporters relative to genome size (Additional file 5), possibly reflecting the difference in ecological niches available to the geographically isolated strains. All genomes were found to encode transporters involved in auxin efflux (Additional file 5); giving further evidence to support the identification of putative IAA production genes in all *P. polymyxa* strains (Table 3)*.* Notably, all *P. polymyxa* strains encode a PstSCAB system (Table 3), a high-affinity, high-velocity phosphate specific transport system [105] suggesting *P. polymyxa* possess a strong capability for phosphate uptake from the environment. A large number of phospho-transferase transporters of the glucose-glucoside family are encoded by all strains. This family of transporters mediates the intake of simple sugars offering a simple transport mechanism for plant derived sugars. In addition to the multitude of simple sugar transport systems, all genomes of *P. polymyxa* encode various transporters involved in the uptake of cellobiose, arabinose, chitobiose and various other plant derived compounds, corroborating the capability of CR1 to degrade and utilize complex plant-derived compounds (our unpublished data). As expected, ABC transporters responsible for export of bacteriocins and other NRPs and PKs are encoded by all strains. In addition, our comparative genomic analysis found all *P. polymyxa* genomes encode a C4-dicarboxylate transporter (*dctA)* (CR1: YP_008909746.1; M1: YP_005957939.1; E681: YP_003868678.1; SC2: YP_003944543.1), reminiscent of *Rhizobia* sp. where dicarboxylic acids (malate, fumarate, and succinate) are major carbon and energy sources for the symbiotic bacteria [70]. In *Rhizobia*, DctD, a C4-dicarboxylate response regulator, activates the expression of *dctA* upon detection of C4-dicarboxylates. Our comparative genomic analysis revealed a *dctD* homolog in CR1 genome (YP_008912290.1), which is absent in the other three *P. polymyxa* genomes.

Consistent with other Gram-positive bacteria, all four *P. polymyxa* genomes encode complete Twin-arginine translocation and Sec systems for protein secretion. Interestingly, all *P. polymyxa* genomes analyzed here appear to encode various genes responsible for type 3 secretion systems (T3SS), however due to the similarity between flagella structures and T3SS structures, it is plausible these genes are actually involved in flagella structure, transport and function (Additional file 5).

## Conclusion

Through detailed comparative analyses we present a global overview of the four completely sequenced *P. polymyxa* genomes and their respective transporters, metabolic pathways, environmental responses, plant-growth promotion traits, biomass degradation, bio-control and bio-product synthesis pathways (Summarized in Figure 11). Despite their geographical isolation and varied plant hosts, the majority of genes implicated in plant association and competitiveness were highly conserved amongst *P. polymyxa* strains. In particular, genes responsible for IAA production, phosphate/phosphonate solubilization, plant cell wall degradation, carbon metabolism and antimicrobial compound production were largely conserved amongst these strains (Table 3, Figure 8). Comparative genomic analyses also identified large numbers of regulatory elements that presumably confer the ability to adapt to diverse environmental cues. In particular, we identified a putative LuxS-dependent quorum sensing system and two possible mechanisms by which *P. polymyxa* senses and adapts to dynamic environmental conditions, σ-factor mediated stress response versus a MCP-mediated chemotactic response.

Not surprisingly minor genome variation exists among *P. polymyxa* strains, as evidenced by the presence of plasmids in M1 and SC2, irregular distribution of strain-specific loci and horizontally transferred genes. Notably, a minimal 9 gene *nif* cluster identified in *P. polymyxa* CR1 is absent in M1, SC2 or E681 (Figure 7). Given the broad host-range of *P. polymyxa* CR1 coupled with a free-living, nitrogen fixation capability, the strain may be developed as a crop inoculant to enhance nitrogen availability. In addition, the *nif* cluster and its regulatory factors may also be developed as a transferable bio-reactor for nitrogen fertilization.

Our study provides new insights into the biology and genome structure of *P. polymyxa*. Our results suggest that the investigated *P. polymyxa* strains have acquired various traits through horizontal gene transfer events. The wide host and geographic range of *P. polymyxa* indicates its intimate relation with host plants and niches, which is consistent with their complex regulatory networks and metabolic versatility. Plant-growth promoting traits identified here offer a framework for further development of *P. polymyxa* strains for agricultural and industrial applications. Knowledge generated from this study will also aid future genetic and metabolic engineering efforts in *P. polymyxa* to enhance their performance in sustainable agricultural practices and renewable energy programs.

## Methods

### Nucleotide accession numbers

The complete genomic sequences of *Paenibacillus polymyxa* strains examined in this study were obtained from Genebank on January 1st, 2014. The accession number of *P. polymyxa* strains are as follows; E681 - [Genebank: NC_014483], M1 - [Genebank: NC_017542], pM1 - [Genebank: NC_017543], SC2 - [Genebank: NC_014622], pSC2 - [Genebank: NC_014628], CR1 - [Genebank: NC_023037].

### Phylogenetic analyses

16S sequences were obtained for publically available *Paenibacillacae* from the NCBI Nucleotide database and aligned using ClustalΩ [106] and manually refined within MEGA6 [44]. For those strains which individual 16S sequences were not available 16S rRNA sequences were obtained from publically available whole genome sequences. From these aligned sequences a phylogeny was generated using the Maximum-likelihood method [43] with default parameters using *Agrobacterium fabrum* C58 as an out-group. Support for the produced phylogenetic tree was determined by performing 1000 bootstrap replications and branches with less than 60% support were collapsed to polytomies. The neighbour joining whole genome phylogeny was generated using the dnadist and neighbour packages in PHYLIP [107] and visualized using phyloXML [108], using tools publically available on the Joint Genome Institute Integrated Microbial Genomes Database [62].

### Genome analysis

Genome annotations of each *P. polymyxa* strain were performed as described previously [27–30]. Annotations were obtained from Genebank on January 1st, 2014. Genomic features were annotated in Artemis [54] and visualized using DNAplotter [55]. General genome features (rRNA, tRNA, and CDS) were identified using the provided annotations from Genebank and the genomic sequences were reanalyzed using tRNAscan [109] and RNAmmer [110]. Information regarding clusters of orthologous groups (COGs), KEGG orthology (KO), protein localization and gene ontology were obtained from the Joint Genome Institute Integrated Microbial Genomes database. Tandem repeats were determined using TandemFinder [111]. Prophage elements and features were identified using PHAST [112] and visualized in Artemis. Insertion sequences were predicted by the IS Finder database [113].

### Genomic island identification

Putative horizontally transferred genes were identified using IslandViewer 2.0 [56], which scans the genome and identifies putative genomic islands by regional differences in GC-content and skew. Genomic islands identified by this method containing greater than 5 genes or larger than 4 kb in size were considered for analysis. Phage related genes contained within putative genomic islands were identified by manual curation of genomic island encoded genes.

### Comparative genomics

The genome of each *P. polymyxa* strain was aligned against other sequenced *P. polymyxa* genomes accessible on Genebank on January 1st, 2014 by determining local collinear blocks (LCBs) using the progressiveMauve algorithm in Mauve [114]. Further alignment was performed using MUMmer [115]. Dot-plots were created by iteratively comparing homologous protein coding sequences using the available tools on the JGI IMG database. Conserved and strain-specific genes were identified using mGenomeSubtractor [52] on default parameters with H-value cut-offs of <0.41 and >0.8 for strain-specific and conserved proteins respectively.

Genes putatively responsible for plant-growth promotion, bio-mass degradation and solventogenesis were identified by using KO and homology searches using tBLASTx to previously characterized homologs. Metabolic and signalling pathways were constructed using the KEGG database [116]. Homologs within these pathways were identified using a cut-off threshold of >50% positive amino acid identity against the closest related available homologue. Encoded transport proteins were identified by BLAST search against the Transporter Classification Database [117] and KO classification [118].

Non-ribosomal peptide synthesis clusters, polyketide clusters and siderophore synthesis genes of both chromosomes and plasmids were identified using antiSMASH [119], and their structure was compared to other known clusters. Homologous proteins between species are identified by use of the same colour. Glucoside hydrolase, pectin lyase, carbohydrate esterase and carbohydrate binding motifs were identified using the CAzY database [82].

Protein homology was determined by performing a BLASTp search against identified homologs. Proteins that met an E-value and positive amino acid identity cut-off of ≤10^−25^ and ≥60%, respectively, were considered homologous.

### Availability of supporting data

All sequences and annotations referenced in the manuscript are publically available on the Genebank Database at the accession numbers provided. Distances used to compute phylogeny are publically available on the Joint Genome Institute’s Integrated Microbial Genomics Database. The data sets supporting the results of this article are included within the article and its additional files.

## Electronic supplementary material

Additional file 1: Figure S1: MUMmer dot-plots comparing protein level homology between completely sequenced *P. polymyxa* genomes. Each strain’s genome is compared pairwise against all other strains. Plasmid comparisons are denoted by their accession numbers and are separated from their respective strains chromosome comparison by a red line. Dots that deviate from the horizontal represent chromosome rearrangements, at the gene level, from the reference genome. The strains listed on the left represent the reference genome for the horizontal row; those listed at the top correspond to the query strain in the vertical column. (TIFF 43 KB)

Additional file 2: **Identified genomic islands and their encoded genes.** (XLSX 53 KB)

Additional file 3: **Identified insertion sequences and their homology to known insertion sequences.** (XLSX 35 KB)

Additional file 4: **Table S1.** CAzY profile of sequenced *P. polymyxa* genomes. **Table S2. σ**-factors and methyl-accepting chemotaxis proteins encoded by *P. polymyxa* strains. **Table S3.** Encoded ABC transporters and PTS family transporter specificities. (DOC 106 KB)

Additional file 5: **Identified non-ribosomal, polyketide and hybrid synthase clusters and their locations.** (XLS 67 KB)

Additional file 6: **Transporter classification homology and the number of identified homologs in the four** ***P. polymyxa*** **strains.** (XLSX 15 KB)

## Abbreviations

PGPR: Plant-growth-promoting rhizobacteria
IAA: Indole-3-acetic acid
CDS: Coding DNA sequences
COG: Clusters of orthologous groups
HGT: Horizontal gene transfer
IS: Insertion sequences
GI: Genomic islands
IPA: Indole-3-pyruvytic-acid
IPDC: Indole-3-pyruvate decarboxylase C
ACC: 1-aminocyclopropane-1-carboxylic acid
GH: Glycoside hydrolase
TCS: Two-component system
MCP: Methyl-accepting chemotaxis protein
QS: Quorum sensing
ABC: ATP-binding cassette.
