# Supplementary figures and images for: Comparative and genetic analysis of the four sequenced Paenibacillus polymyxa genomes reveals a diverse metabolism and conservation of genes relevant to plant-growth promotion and competitiveness
Source: BMC Genomics. 2014 Oct 3;15:851. doi: 10.1186/1471-2164-15-851 (PMC4209062; doi:10.1186/1471-2164-15-851)

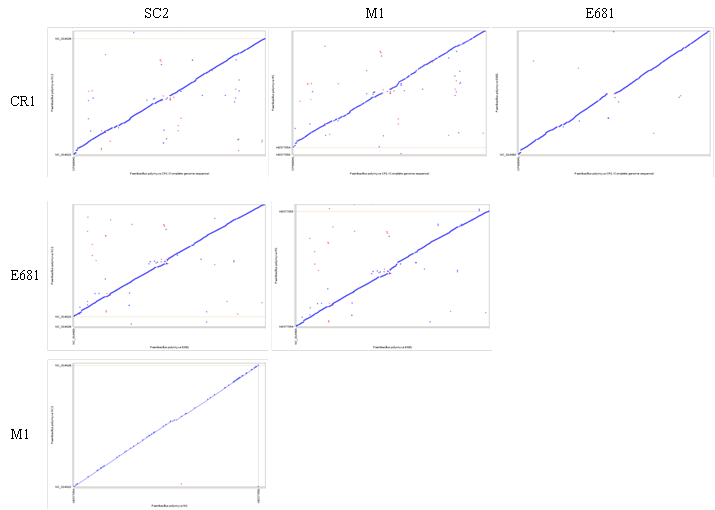

Supplement: Supplementary file 1 — Additional file 1: Figure S1: MUMmer dot-plots comparing protein level homology between completely sequenced P. polymyxa genomes. Each strain’s genome is compared pairwise against all other strains. Plasmid comparisons are denoted by their accession numbers and are separated from their respective strains chromosome comparison by a red line. Dots that deviate from the horizontal represent chromosome rearrangements, at the gene level, from the reference genome. The strains listed on the left represent the reference genome for the horizontal row; those listed at the top correspond to the query strain in the vertical column. (TIFF 43 KB) [file 12864_2014_6587_MOESM1_ESM.tiff]
