# Supplementary material for: Comparative and genetic analysis of the four sequenced Paenibacillus polymyxa genomes reveals a diverse metabolism and conservation of genes relevant to plant-growth promotion and competitiveness
Source: BMC Genomics. 2014 Oct 3;15:851. doi: 10.1186/1471-2164-15-851 (PMC4209062; doi:10.1186/1471-2164-15-851)
Supplement: Supplementary file 4 — Additional file 4: Table S1. CAzY profile of sequenced P. polymyxa genomes. Table S2. σ-factors and methyl-accepting chemotaxis proteins encoded by P. polymyxa strains. Table S3. Encoded ABC transporters and PTS family transporter specificities. (DOC 106 KB) [file 12864_2014_6587_MOESM4_ESM.doc]

| **Supplementary Table 1. CAzY profile of sequenced *P. polymyxa* genomes** | | | | | | | | | |
| --- | --- | --- | --- | --- | --- | --- | --- | --- | --- |
| GH Family | Number of CDS | | | | PL Family | Number of CDS | | | |
|  | E681 | M1 | SC2 | CR1 |  | E681 | M1 | SC2 | CR1 |
| 1 | 8 | 10 | 8 | 17 | 1 | 4 | 3 | 3 | 5 |
| 2 | 4 | 4 | 4 | 7 | 3 | 1 | 1 | 1 | 1 |
| 3 | 4 | 4 | 4 | 7 | 9 | 2 | 2 | 2 | 1 |
| 4 | 3 | 3 | 3 | 3 | 10 | 1 | 1 | 1 | 1 |
| 5 | 7 | 7 | 7 | 5 | 11 | 11 | 2 | 2 | 2 |
| 6 | 1 | 1 | 1 | 1 | NC | 0 | 1 | 1 | 0 |
| 10 | 1 | 3 | 1 | 2 | CE Family | Number of CDS | | | |
| 11 | 1 | 2 | 1 | 2 |  | E681 | M1 | SC2 | CR1 |
| 13 | 9 | 9 | 9 | 10 | 1 | 2 | 3 | 2 | 3 |
| 14 | 1 | 1 | 1 | 1 | 2 | 1 | 1 | 1 | 1 |
| 16 | 2 | 3 | 2 | 1 | 4 | 15 | 13 | 12 | 13 |
| 18 | 3 | 3 | 3 | 3 | 7 | 2 | 2 | 2 | 2 |
| 23 | 2 | 3 | 2 | 3 | 8 | 2 | 2 | 2 | 2 |
| 24 | 1 | 1 | 1 | 0 | 9 | 1 | 1 | 1 | 1 |
| 25 | 2 | 3 | 2 | 3 | 12 | 4 | 4 | 4 | 4 |
| 26 | 5 | 5 | 5 | 4 | 14 | 1 | 1 | 1 | 1 |
| 27 | 1 | 1 | 1 | 1 | CBM Family | Number of CDS | | | |
| 28 | 1 | 1 | 1 | 1 |  | E681 | M1 | SC2 | CR1 |
| 30 | 2 | 2 | 2 | 2 | 3 | 5 | 5 | 5 | 4 |
| 31 | 1 | 1 | 1 | 1 | 6 | 2 | 2 | 2 | 2 |
| 32 | 10 | 10 | 10 | 9 | 12 | 0 | 0 | 0 | 1 |
| 35 | 1 | 1 | 1 | 1 | 13 | 1 | 3 | 3 | 2 |
| 36 | 4 | 4 | 4 | 3 | 16 | 0 | 1 | 1 | 0 |
| 38 | 0 | 0 | 0 | 1 | 22 | 1 | 2 | 2 | 2 |
| 42 | 3 | 3 | 3 | 5 | 25 | 2 | 2 | 1 | 2 |
| 43 | 10 | 10 | 10 | 9 | 26 | 0 | 1 | 0 | 1 |
| 44 | 1 | 1 | 1 | 1 | 32 | 2 | 3 | 3 | 2 |
| 46 | 1 | 1 | 1 | 1 | 34 | 1 | 1 | 1 | 1 |
| 48 | 1 | 1 | 1 | 0 | 35 | 4 | 3 | 3 | 1 |
| 51 | 3 | 3 | 3 | 3 | 36 | 2 | 3 | 2 | 3 |
| 52 | 1 | 1 | 1 | 2 | 38 | 0 | 3 | 3 | 1 |
| 53 | 1 | 1 | 1 | 2 | 41 | 1 | 1 | 1 | 1 |
| 65 | 1 | 1 | 1 | 1 | 46 | 1 | 1 | 1 | 1 |
| 67 | 1 | 1 | 1 | 1 | 48 | 2 | 2 | 2 | 2 |
| 68 | 1 | 1 | 1 | 1 | 50 | 3 | 6 | 4 | 7 |
| 74 | 1 | 1 | 1 | 1 | 56 | 1 | 1 | 0 | 0 |
| 78 | 1 | 1 | 1 | 3 | 59 | 0 | 1 | 1 | 0 |
| 81 | 1 | 1 | 1 | 0 | 61 | 0 | 0 | 0 | 1 |
| 84 | 1 | 1 | 1 | 1 | 63 | 0 | 1 | 1 | 1 |
| 88 | 1 | 1 | 1 | 1 | 66 | 3 | 3 | 3 | 7 |
| 94 | 2 | 2 | 2 | 3 |  |  |  |  |  |
| 95 | 1 | 1 | 1 | 1 |  |  |  |  |  |
| 105 | 3 | 3 | 3 | 3 |  |  |  |  |  |
| 112 | 1 | 1 | 1 | 1 |  |  |  |  |  |
| 115 | 1 | 1 | 1 | 1 |  |  |  |  |  |
| 120 | 0 | 0 | 0 | 1 |  |  |  |  |  |
| 127 | 1 | 1 | 1 | 1 |  |  |  |  |  |
| 130 | 2 | 2 | 2 | 2 |  |  |  |  |  |
| NC | 1 | 1 | 1 | 0 |  |  |  |  |  |
| Data was obtained from the CAzY Database. GH – Glycoside Hydrolase, PL – Pectin Lyase, CE- Carbohydrate Esterase, CBM – Carbohydrate Binding Motif | | | | | | | | | |

| **Supplementary Table 2. σ-factors and methyl-accepting chemotaxis proteins encoded by *P. polymyxa* strains** | | | | | |
| --- | --- | --- | --- | --- | --- |
| Genome | COG0568 | COG1191 | COG1508 | COG1595 | COG0840 |
| *Paenibacillus polymyxa* CR1 | 1 | 7 | 1 | 15 | 24 |
| *Paenibacillus polymyxa* E681 | 1 | 6 | 1 | 13 | 27 |
| *Paenibacillus polymyxa* M1 | 2 | 6 | 1 | 22 | 25 |
| *Paenibacillus polymyxa* SC2 | 1 | 7 | 1 | 20 | 25 |
| COG categories contain the following genes; COG0568 - rpoD, COG1191 - sigE and sporulation factors, COG1508 - rpoN, COG1595 - extracellular sigma factors, COG0840- methyl-accepting chemotaxis proteins. COG categorization was performed using available tools on the JGI IMG database | | | | | |

| **Supplementary Table 3. Encoded ABC transporters and PTS family transporter specificities** | | | | | | | | | | |
| --- | --- | --- | --- | --- | --- | --- | --- | --- | --- | --- |
|  |  | **ATP-binding cassette** | | | | | | | |  |
|  | ***Strain*** | **Ion** | **Sugars** | | **Phosphate/aa** | | **Peptide/nickel** | **Metallic cation** | **ABC-2** |  |
|  | **E681** | Sulfate, molybdate, iron(III), spermidine/putrescine, glycine betaine/proline, | raffinose/stachyose/melibiose, lactose/L-arabinose, alduronate, cellobiose, chitobiose, arabinooligiosaccharide, multisugar, ribose/xylose, G-3-P | | phosphate, phosphonate, glutamine, cysteine x2, methionine, | | oligopeptide, nickel, | iron complex, zinc, zinc/manganese, iron(II), biotin | lipopolysaccharide, bacitracin, cell div, techioic acid |  |
|  | **M1** | Sulfate, molybdate, iron(III), spermidine/putrescine, | raffinose/stachyose/melibiose, lactose/L-arabinose, alduronate, cellobiose, chitobiose, arabinooligiosaccharide, multisugar, ribose/xylose, G-3-P | | phosphate, phosphonate, glutamine, cysteine x2, methionine, | | oligopeptide, nickel, | iron complex, zinc, zinc/manganese, iron(II), biotin | lipopolysaccharide, bacitracin, cell div, techioic acid |  |
|  | **SC2** | Sulfate, molybdate, iron(III), spermidine/putrescine, glycine betaine/proline, | raffinose/stachyose/melibiose, lactose/L-arabinose, alduronate, cellobiose, chitobiose, arabinooligiosaccharide, multisugar, ribose/xylose | | phosphate, phosphonate, glutamine, cysteine x2, methionine, | | oligopeptide, nickel, | iron complex, zinc, zinc/manganese, iron(II), biotin | lipopolysaccharide, bacitracin, cell div, techioic acid |  |
|  | **CR1** | Sulfate, molybdate, iron(III), spermidine/putrescine, glycine betaine/proline, osmoprotectant | raffinose/stachyose/melibiose, lactose/L-arabinose, alduronate, cellobiose, chitobiose, arabinooligiosaccharide, multisugar, ribose/xylose, G-3-P | | phosphate, phosphonate, glutamine, cysteine x2, methionine, | | oligopeptide, nickel, | iron complex, zinc, zinc/manganese, iron(II), biotin | lipopolysaccharide, bacitracin, cell div, techioic acid |  |
|  |  | **PTS Family** | | | | | |  |  |  |
|  | ***Strain*** | **GLC** | **LAC** | **FRU** | | **MAN** | |  |  |  |
|  | **E681** | glucose, n-acetyl-d-glucosamine, d-glucosamine, sucrose, b-glucoside | cellobiose | mannitol, fructose | | mannose | |  |  |  |
|  | **M1** | glucose, n-acetyl-d-glucosamine, d-glucosamine, sucrose, b-glucoside | cellobiose | mannitol, fructose | | mannose | |  |  |  |
|  | **SC2** | glucose, n-acetyl-d-glucosamine, d-glucosamine, sucrose, b-glucoside | cellobiose | mannitol, fructose | | mannose | |  |  |  |
|  | **CR1** | glucose, n-acetyl-d-glucosamine, d-glucosamine, sucrose, b-glucoside | cellobiose | mannitol, fructose | | mannose | |  |  |  |
|  | ABC transporter and PTS family transporter specificities were obtained using KO in combination with Transporter Classification Database classification system. | | | | | | |  |  |  |
